# SARIFA and Lipid Metabolic Reprogramming in Prostate Cancer: Fundamental Mechanisms, Tumor Microenvironment, and Novel Biomarker Prospects

**DOI:** 10.3390/life16081304

**Published:** 2026-08-09

**Authors:** Liudmila Mikhaleva, Maria Martynova, Zarina Gioeva, Nikolay Shakhpazyan, Nikita Chizhikov, Valentina Pechnikova, Mikhail Gushchin, Alexander Ilyichev

**Affiliations:** 1 Petrovsky National Research Centre of Surgery, 117418 Moscow, Russia; mikhalevalm@yandex.ru (L.M.); nshakhpazyan@gmail.com (N.S.); valiagtx@yandex.ru (V.P.); guschin.michail@yandex.ru (M.G.); alex730110@gmail.com (A.I.); 2 State Budgetary Healthcare Institution “Republican Pathology Bureau” of the Ministry of Health of the Republic of North Ossetia—Alania, 362003 Vladikavkaz, Russia; mariamartynova816@gmail.com; 3 Moscow Botkin Multidisciplinary Scientific-Clinical Center, 125284 Moscow, Russia

**Keywords:** prostate cancer, SARIFA, adipocytes, biomarker, lipid metabolic reprogramming, tumor microenvironment

## Abstract

Prostate cancer (PCa) is one of the most prevalent malignancies in men worldwide. Established clinicopathological parameters do not fully describe the biological heterogeneity of PCa or consistently predict its clinical course after radical prostatectomy, supporting the investigation of additional prognostic markers. Stroma AReactive Invasion Front Areas (SARIFA) is a recently described histomorphological pattern characterized by direct contact between tumor cells and adipocytes without intervening desmoplastic or inflammatory stroma at the invasion front. Limited retrospective evidence from prostatectomy specimens indicates that SARIFA positivity is associated with adverse pathological features. However, its independent prognostic value and clinical utility remain to be established. Because SARIFA can potentially be assessed on routinely prepared hematoxylin and eosin-stained sections without additional molecular assays or immunohistochemical staining, it represents a potentially accessible candidate marker, although its reproducibility, standardization, and cost-effectiveness require formal evaluation. This review summarizes current evidence concerning lipid metabolic remodeling and tumor–adipocyte interactions that may contribute to the SARIFA phenotype in PCa. Androgen receptor-regulated lipid metabolism, uptake of adipocyte-derived fatty acids, adipokine signaling, and extracellular vesicle-mediated communication provide a plausible mechanistic framework. Nevertheless, direct experimental evidence linking several of these processes specifically to SARIFA in PCa remains limited, and some proposed relationships are based on indirect evidence or findings from other tumor types. Further studies should establish standardized scoring criteria, intra- and interobserver reproducibility, external validation, and incremental prognostic value before the clinical implementation of SARIFA can be considered.

## 1. Introduction

Prostate cancer (PCa) is one of the most frequently diagnosed malignant tumors in men worldwide and remains a major cause of cancer-related mortality. It is the most commonly diagnosed male cancer in more than half of the world’s countries, and the annual number of newly diagnosed cases has been projected to increase from approximately 1.4 million in 2020 to 2.9 million by 2040 because of population growth, aging, and increasing detection [1,2,3]. Although many patients with localized PCa can be treated with curative intent, disease recurrence, metastatic progression, and the development of castration-resistant prostate cancer remain major clinical challenges [4,5].

Risk stratification in PCa is based on established clinical and pathological parameters, including serum prostate-specific antigen levels, histological Grade, tumor stage, lymph node status, and surgical margin status. These variables provide clinically important prognostic information but do not fully account for the biological and morphological heterogeneity of the disease. Consequently, additional tissue-based features are being investigated to determine whether they can improve postoperative risk stratification when used together with established parameters [5].

Within this biomarker landscape, lipid-metabolic proteins and morphological correlates of tumor–adipose interaction remain investigational and are not incorporated into established PCa risk-classification systems. Their potential value would therefore lie in providing information complementary to, rather than replacing, PSA, histological grading, pathological staging, and selectively used genomic classifiers.

Stroma AReactive Invasion Front Areas (SARIFA) is a recently described histomorphological pattern characterized by direct contact between tumor cells and adipocytes without intervening desmoplastic or inflammatory stroma at the invasion front [6,7]. SARIFA can be assessed on routinely prepared hematoxylin and eosin-stained sections and has been associated with adverse outcomes in several tumor types [6,7,8,9,10,11]. However, evidence in PCa remains limited and is currently derived predominantly from a retrospective analysis of radical prostatectomy specimens from patients with pT3a disease [12]. In that cohort, SARIFA positivity was associated with adverse clinicopathological characteristics, whereas the observed differences in survival outcomes did not reach statistical significance [12]. Therefore, SARIFA should currently be regarded as a candidate histomorphological marker rather than a clinically validated prognostic biomarker in PCa.

Conceptually, SARIFA differs from established clinicopathological and molecular prognostic tools. The Gleason score and ISUP Grade Group characterize tumor architectural differentiation, whereas pathological stage, extraprostatic extension, lymphovascular invasion, surgical margin status, and lymph node involvement describe anatomical disease extent and routes of spread. Invasive cribriform carcinoma and intraductal carcinoma represent adverse architectural patterns recommended for pathological reporting, while the independent clinical significance of perineural invasion and the assessment of tumor budding are less consistently established [5,13,14]. Tissue-based genomic classifiers, including Decipher, provide tumor-intrinsic molecular risk estimates and may be used selectively when their results are expected to affect clinical management [14,15]. SARIFA instead identifies a spatially restricted, stroma-areactive tumor–adipose interface at the invasive front. Its potential novelty lies in capturing a morphological correlate of tumor–adipose interaction and associated metabolic and microenvironmental remodeling on routine H&E-stained sections. This conceptual distinction does not establish independent or incremental prognostic value, which must be tested directly against established clinicopathological variables and emerging biomarkers.

The biological processes associated with the SARIFA phenotype remain incompletely understood. Available findings indicate that androgen receptor-regulated lipid metabolism, interactions between tumor cells and periprostatic adipose tissue, adipokine signaling, fatty-acid uptake, and extracellular vesicle-mediated communication may contribute to metabolic and microenvironmental conditions compatible with the SARIFA morphology [7,12,16,17,18,19,20,21]. Nevertheless, direct experimental evidence linking several of these processes specifically to SARIFA in PCa remains limited. Some proposed relationships are based on prostate cancer models in which SARIFA status was not assessed, whereas others have been extrapolated from SARIFA studies in different tumor types.

Previous reviews have considered SARIFA primarily as a pan-cancer histomorphological phenomenon or have focused on tumor entities in which the clinical evidence is most developed, particularly gastric and colorectal cancers [7]. A PCa-centered synthesis integrating SARIFA morphology with the distinctive lipid metabolism of the prostate, periprostatic adipose tissue, extracellular-vesicle signaling, immune regulation, and computational pathology remains lacking. An additional unresolved issue is the distinction between findings demonstrated directly in SARIFA-positive PCa and mechanisms inferred from PCa models without SARIFA assessment or from other cancer types. The present review was designed to address this gap. The aim of this review is to critically summarize the available evidence concerning SARIFA in PCa, with particular emphasis on tumor–adipocyte interactions, lipid metabolic remodeling, and the tumor microenvironment. We also discuss the limitations of the current evidence, distinguish experimentally demonstrated findings from mechanistic hypotheses, and outline the validation requirements that should be addressed before the potential clinical utility of SARIFA can be determined. The literature-search approach and hierarchy used to interpret direct and indirect evidence are described below.

### Literature Search and Evidence Synthesis

This narrative review was informed by a structured literature search conducted in PubMed/MEDLINE and PubMed Central, supplemented by targeted searches of journal and publisher websites and web-based searches for recently published studies, clinical guidelines, and consensus documents. The search covered the period from database inception to 2 August 2026. Search terms included “SARIFA,” “Stroma AReactive Invasion Front Areas,” “prostate cancer,” “tumor–adipose interface,” “periprostatic adipose tissue,” “lipid metabolism,” “fatty-acid metabolism,” “extracellular vesicles,” “exosomes,” “immune microenvironment,” “tumor-adipose feature,” “artificial intelligence,” and related combinations. Reference lists of relevant primary studies and review articles were also examined to identify additional publications. Original studies directly evaluating SARIFA were considered together with mechanistic, translational, and methodological studies relevant to lipid metabolism, tumor–adipose interactions, extracellular vesicles, immune regulation, and computational pathology. Primary SARIFA-specific studies in PCa were assigned the greatest evidentiary weight. Studies of PCa in which SARIFA status was not assessed were used only to establish mechanistic plausibility, whereas SARIFA studies in gastrointestinal and pancreatic cancers were used to identify potentially relevant biological relationships requiring independent validation in PCa. Reviews, consensus documents, and clinical guidelines were used for contextual and methodological purposes. Owing to the limited number and methodological heterogeneity of PCa-specific studies, no formal meta-analysis was performed.

## 2. The SARIFA Phenomenon: A Morphological Feature Potentially Associated with Metabolic Reprogramming

SARIFA was first described in 2021 as a histomorphological feature. It is characterized by the direct contact between tumor cells and adipocytes without intervening collagenous or inflammatory stroma at the tumor invasion front. SARIFA can be assessed on routinely available H&E-stained slides by standard light microscopy without additional staining or molecular testing [7]. Associations between SARIFA positivity and adverse clinical outcomes have been reported in several independent cohorts of patients with colorectal, gastric, pancreatic, and esophagogastric cancers [6,7,8,9,10,11]. In PCa, however, the available evidence is currently limited to a single retrospective cohort of 301 patients with pT3a tumors treated by radical prostatectomy [12]. In gastric and colorectal cancer, SARIFA was defined as the direct contact between a cluster of tumor glands/cells comprising at least five tumor cells and inconspicuous surrounding adipose tissue at the invasion front [6].

Evidence specific to prostate cancer. In PCa, direct SARIFA-specific evidence is currently limited to the retrospective study by Enke et al., which included 301 patients with pT3a tumors treated by radical prostatectomy [12]. SARIFA positivity was significantly associated with microscopic residual tumor/positive surgical margin status, non-focal extraprostatic extension, and higher Gleason scores and Grade Groups [12]. Furthermore, SARIFA-positive PCas tended to have a higher frequency of lymphatic invasion. Notably, when comparing the number of positive lymph nodes among all pN1 PCa cases, SARIFA-positive PCa cases exhibited a higher absolute count of positive lymph nodes (*p* = 0.008), even though the number of dissected lymph nodes did not differ between SARIFA-positive and SARIFA-negative cases (*p* = 0.439) [12]. The expression levels of fatty-acid-binding protein 4 (FABP4) and the fatty-acid translocase CD36, two key proteins involved in cellular lipid metabolism, were assessed in the same study. While no SARIFA-dependent differences were observed in FABP4 protein expression (*p* = 0.53), SARIFA-positive cases exhibited a statistically significant increase in CD36 expression in the tumor cells of the invasion front (*p* = 0.00014). No differences regarding CD36 expression were observed in the cells of the tumor center (*p* = 0.17) [12].

Several studies have demonstrated that SARIFA-positive status significantly correlates with progression-free survival and increased rates of adverse events [6,8,9,10,11].

Evidence from gastrointestinal cancers. In contrast to PCa, SARIFA has been evaluated in several independent cohorts of patients with colorectal, gastric, pancreatic, and esophagogastric cancers, where associations with recurrence-free, progression-free, cancer-specific, or overall survival have been reported [6,7,8,9,10,11]. These studies provide important evidence concerning the broader biological and prognostic context of SARIFA but should not be interpreted as direct validation of SARIFA in PCa. In a recent colorectal cancer study, Reitsam et al. investigated the relationship between SARIFA status and stromal morphometry in 164 patients of colorectal cancer (CRC) patients, as well as its association with the recurrence-free survival (RFS), luminal proportion of tumor (PoT) [22]. In a cohort of 164 CRC resection specimens, Reitsam et al. found that SARIFA positivity was independently associated with shorter recurrence-free survival in a multivariable Cox model adjusted for pT category, pN category, grade of differentiation, and PoT (adjusted HR, 2.595; 95% CI, 1.085–6.205; *p* = 0.032). Among patients with locally advanced pT3/pT4 CRC, SARIFA-positive cases (n = 36) also showed shorter recurrence-free survival (HR, 2.63; 95% CI, 1.371–5.046; *p* = 0.0026) [22]. SARIFA positivity was also associated with a lower luminal proportion of tumor, corresponding to a higher stromal content (*p* = 0.009), and with lower vessel density at the luminal tumor component (*p* = 0.0059) [22]. These morphometric associations should be regarded as secondary findings and require independent validation.

There was a relationship between the SARIFA-status measured at the invasion front and proportion of tumor measured at the luminal tumor surface: SARIFA-positive CRCs were more often found to be luminal PoT-low (e.g., stroma proportion high) (*p* = 0.009) [22]. This paradox raises a fundamental issue concerning the biology of SARIFA-positive tu-mors: why tumor cells with a higher stroma content in the center, do not induce a desmo-plastic stroma reaction when contacting adipocytes at the invasion front? It was also pointed out that SARIFA-positive CRCs were characterized by a lower vessel content at the luminal tumor component (*p* = 0.0059) [22].

The association between SARIFA status at the invasion front and luminal tumor composition in CRC may be relevant to the future development of surrogate morphological markers [10]. However, no comparable surrogate approach has been validated in PCa. Because direct SARIFA assessment requires visualization of tumor cells in contact with periprostatic adipose tissue, routine prostate needle biopsy specimens may not reliably capture the relevant tumor–adipose interface. At present, SARIFA assessment in PCa is therefore primarily applicable to radical prostatectomy specimens [12], while its feasibility in biopsy or other limited tissue samples requires dedicated investigation. Recent studies in gastric and colorectal cancers have associated SARIFA positivity with altered tumor-cell lipid metabolism and immune responses [6,23]. These findings provide a biological rationale for investigating analogous mechanisms in PCa but cannot establish the prevalence, prognostic significance, molecular characteristics, or clinical utility of SARIFA in prostate cancer.

### 2.1. Limitations of the Current Evidence for SARIFA in Prostate Cancer

The current evidence concerning SARIFA is methodologically heterogeneous. Published studies differ in tumor type, disease stage, treatment setting, cohort size, clinical endpoint, tissue sampling, and assessment approach [6,7,8,9,10,11,12]. Most studies are retrospective, and the evaluated outcomes range from clinicopathological characteristics to recurrence-free, cancer-specific, and overall survival. Accordingly, findings obtained in gastrointestinal and pancreatic cancers cannot be directly extrapolated to PCa.

In PCa, direct clinical evidence is currently limited to a single retrospective study of 301 patients with pT3a tumors treated by radical prostatectomy [12]. Subsequent reviews have discussed this study without adding an independent PCa cohort [24]. SARIFA positivity was associated with positive surgical margins, non-focal extraprostatic extension, and higher Gleason scores and Grade Groups, whereas differences in overall survival and biochemical recurrence-free survival did not reach statistical significance [12]. Analyses of biochemical recurrence and PSMA-PET/CT findings were restricted to substantially smaller patient subgroups, limiting the precision and generalizability of these findings [12]. For several clinicopathological comparisons, the primary PCa study reported statistical significance predominantly through *p* values without consistently providing effect estimates and confidence intervals, which limits assessment of the magnitude and precision of the observed associations.

The study used whole-mount, large-format histology. Binary SARIFA positivity was defined by the presence of at least one positive area, whereas an additional quantity-dependent classification used positivity in at least one-third of the examined slides [12]. Because this quantitative cut-off was derived from the same cohort, it requires independent external validation. SARIFA classification may also depend on the extent of tissue sampling, the number of examined sections, and the selected positivity threshold. Favorable interobserver agreement has been reported in colorectal cancer [8]. However, in the PCa study, one investigator performed the initial assessment, while ambiguous cases were reviewed jointly with a board-certified pathologist [12], formal PCa-specific analyses of interobserver and intraobserver reproducibility were not reported. Other potential sources of bias include the single-center design, restriction to surgically treated pT3a tumors, limited availability of outcome data, and possible publication bias in a small literature dominated by positive associations.

The principal limitation is therefore the absence of independent PCa cohorts rather than inconsistency among multiple prostate cancer studies.

The remaining limitations can be grouped into four principal domains. The limitations of the current evidence can be grouped into four principal domains. First, the clinical evidence is restricted to a single retrospective cohort of surgically treated patients with pT3a disease, limiting external validity and preventing assessment across the broader spectrum of PCa. Second, SARIFA assessment requires an adequately sampled tumor–adipose interface, and its feasibility in routine needle-biopsy specimens remains unknown. Third, scoring may depend on section sampling, the selected positivity threshold, and pathologist interpretation, while standardized training and formal PCa-specific reproducibility studies are lacking. Fourth, the biological mechanisms proposed to underlie SARIFA are based largely on indirect evidence from PCa models without SARIFA assessment or from gastrointestinal cancers. Consequently, the independent prognostic value, predictive utility, clinical impact, and cost-effectiveness of SARIFA remain undetermined, and its use should currently be restricted to research settings.

### 2.2. Clinical Outcome and Treatment-Response Evidence

Direct evidence linking SARIFA to clinical outcomes in PCa remains limited to the retrospective pT3a radical prostatectomy cohort reported by Enke et al. [12]. In the full cohort, SARIFA-positive tumors showed a directionally poorer overall survival, but the association was not statistically significant (HR, 1.290; 95% CI, 0.758–2.196; *p* = 0.35). A quantity-dependent definition of SARIFA positivity, requiring positive areas in at least one-third of the examined slides, similarly showed a non-significant association with overall survival (HR, 1.547; 95% CI, 0.832–2.876; *p* = 0.16). In the reported exploratory analysis of biochemical recurrence-free survival involving 48 patients, separation of the survival curves was also observed without statistical significance (HR, 1.632; 95% CI, 0.883–3.015; *p* = 0.11) [12]. Therefore, the available data do not establish SARIFA as an independently validated prognostic marker for overall survival or biochemical recurrence-free survival in PCa.

The same study included an exploratory analysis of PSMA-PET/CT findings in a selected subgroup of 57 patients [12]. Local recurrence rates did not differ according to SARIFA status (*p* = 0.877). SARIFA-positive cases showed numerically higher frequencies of regional lymph node involvement, distant lymph node metastases, and bone metastases; however, none of these comparisons reached statistical significance (*p* = 0.140, *p* = 0.066, and *p* = 0.061, respectively). These findings describe the anatomical distribution of PSMA-positive recurrence in a small, retrospectively selected subgroup and should not be interpreted as evidence that SARIFA predicts metastasis-free survival or defines a validated metastatic phenotype.

Metastasis-free survival and prostate cancer-specific survival have not been evaluated according to SARIFA status. Moreover, no published study has assessed whether SARIFA is associated with response to androgen deprivation therapy, androgen receptor pathway inhibitors, radiotherapy, chemotherapy, immunotherapy, or other systemic treatments. Current evidence therefore concerns a possible prognostic association and does not establish predictive utility. Demonstration of a treatment-predictive biomarker requires comparison of treatment effects between biomarker-defined groups and formal evaluation of a treatment-by-biomarker interaction, preferably in prospective or randomized studies [25]. Until such evidence becomes available, SARIFA status should not be used to select or modify treatment.

The currently available primary PCa-specific evidence on SARIFA is summarized in Table 1. To date, only one primary study has evaluated SARIFA in PCa; subsequent reviews have discussed the same dataset without providing an independent validation cohort.

## 3. Lipid Metabolism in Normal Prostate Tissues

Normal human prostate epithelial cells uniquely accumulate high intracellular zinc concentrations [26]. Zinc inhibits mitochondrial aconitase, restricting the conversion of citrate to isocitrate and functionally truncating the tricarboxylic acid cycle. This promotes citrate accumulation and secretion at the cost of reduced energy production from citrate oxidation [27]. During malignant transformation, reduced intracellular zinc accumulation relieves this inhibition, allowing increased citrate oxidation and more complete tricarboxylic acid-cycle activity. Thus, early PCa differs from many other malignancies in that malignant transformation initially restores a previously restricted oxidative pathway rather than simply replacing oxidative metabolism with glycolysis [26,27,28,29].

The androgen receptor (AR) regulates de novo lipogenesis by driving the expression of key lipogenic enzymes, including ATP-citrate-lyase (ACLY), fatty acid synthase (FASN), and acetyl-CoA-carboxylase (ACAC). This androgen-driven process is essential for synthesizing cholesterol and fatty acids, which are significant components of the prostate fluid [16,30]. Recent studies of androgen deprivation in castrated rats and primates demonstrate a marked decline in lipid synthesis, re-emphasizing the unique metabolism of the prostate gland. This distinctive metabolic pathway, essential for supporting reproductive function, sets it apart from other tissues [28,31].

Peroxisomal lipid metabolism provides an additional link between prostate biology and PCa-associated metabolic reprogramming. Alpha-methylacyl-CoA racemase (AMACR/P504S) is a mitochondrial and peroxisomal enzyme involved in the metabolism of 2-methyl-branched-chain fatty-acid and bile-acid intermediates. By converting branched-chain acyl-CoA substrates into stereoisomers compatible with subsequent β-oxidation, AMACR facilitates their catabolism. AMACR is markedly overexpressed in prostatic adenocarcinoma and is widely used as an adjunct immunohistochemical marker together with morphological assessment and basal-cell markers [32]. Other components of the peroxisomal branched-chain fatty-acid β-oxidation pathway, including D-bifunctional protein and ACOX3, are also upregulated in PCa, indicating that this pathway represents a biological feature of altered lipid catabolism rather than only a diagnostic epiphenomenon [33]. However, neither AMACR expression nor peroxisomal β-oxidation has been investigated according to SARIFA status.

## 4. Lipid Metabolism in Prostate Cancer

Accumulating evidence indicates that lipid metabolic reprogramming contributes to prostate cancer development and progression by supporting cellular energy demands and metastatic adaptation [34,35,36]. These findings provide a biological context for investigating SARIFA but do not establish a direct mechanistic link between lipid metabolic reprogramming and the SARIFA phenotype.

At the clinical level, Hu et al. retrospectively analyzed 520 men who underwent prostate biopsy and reported that higher circulating low-density lipoprotein cholesterol levels were associated with PCa detection, higher Gleason scores, and more advanced TNM stages [37]. These findings provide additional clinical support for an association between altered lipid status and PCa aggressiveness. However, the retrospective design does not establish causality, and circulating LDL cholesterol was not evaluated in relation to tumor lipid metabolism, the tumor–adipose interface, or SARIFA status.

PCa is characterized by a unique metabolic profile that distinguishes it from most solid tumors. While many malignancies exhibit a shift to aerobic glycolysis (the Warburg effect), early-stage PCa cells maintain a reliance of mitochondrial oxidative phosphorylation and the late-tricarboxylic acid (TCA) cycle for energy production. PCa cells acquire metabolic plasticity by switching to enhanced aerobic glycolysis and activating lipid metabolic reprogramming [34]. Whether these metabolic changes directly contribute to the formation of the SARIFA phenotype remains unknown. Androgen receptor (AR) signaling plays a central role in modulating lipid metabolic processes. In a normal prostate, lipid metabolism is also controlled by sterol regulatory element-binding proteins (SREBPs), specifically SREBP1 and SREBP2, which are transcription factors that regulate the synthesis of fatty acids and cholesterol. Tumor cells increase cholesterol synthesis through SREBP-2 activation to support membrane formation and intratumoral androgen synthesis [38,39,40]. During tumorigenesis, lipid metabolism reprogramming is initiated mainly through the upregulation of SREBP1, ACLY, ACAC, and FASN driven by AR signaling [34]. As demonstrated by Huang et al., SREBP-1 overexpression was positively associated with the clinical Gleason grades in human prostate cancer (*p* = 0.003), promoting increased AR signaling, lipid accumulation in the cells, and FASN expression. Conversely, SREBP-1 knockdown reversed these effects in prostate cancer cell lines (C4–2B), resulting in a marked decrease in these processes. In vivo xenograft model using SREBP-1-overexpressing human LNCaP prostate cancer cells implanted into castrated nude mice established that SREBP-1 induces the castration-resistant progression of prostate cancer cells. This process is driven by an AR-SREBP-1 molecular axis, which constitutes a self-regulating cycle that maintains the continuous expression of transcription factor genes [35].

The findings demonstrate that FASN is further upregulated during prostate cancer progression and castration-resistant form development, contributing to poor prognosis and resistance to hormone therapy [35]. There is also evidence that FASN affects tumorigenesis, tumor cell migration, and proliferation, and blocks apoptosis [36,41].

Beyond the AR–SREBP–FASN axis, lipid metabolic plasticity in PCa involves coordinated regulation of fatty-acid synthesis, desaturation, uptake, intracellular transport, storage, and oxidation. ACAC/ACC produces malonyl-CoA for de novo fatty-acid synthesis, whereas stearoyl-CoA desaturase 1 (SCD1) generates monounsaturated fatty acids that contribute to membrane remodeling, lipid storage, and adaptation to metabolic stress. CD36 mediates the uptake of exogenous fatty acids, while fatty-acid-binding proteins, including FABP4, regulate their intracellular transport and compartmentalization. Carnitine palmitoyltransferase 1A (CPT1A) controls the mitochondrial entry of long-chain fatty acids and thereby represents a major regulatory step in fatty-acid β-oxidation. Peroxisome proliferator-activated receptors (PPARs), particularly PPARγ, function as lipid-sensitive nuclear receptors and have been implicated in context-dependent transcriptional and metabolic programs during PCa progression [34,42,43].

Lipid mobilization in PCa is also regulated by monoacylglycerol lipase (MAGL), which hydrolyzes monoacylglycerols to glycerol and free fatty acids and thereby contributes to the generation of bioactive lipid-signaling molecules. MAGL was initially shown to be highly expressed in aggressive cancer cells and to regulate a fatty-acid network supporting migration, invasion, survival, and tumor growth [44]. In PCa models, MAGL functions together with FASN and fatty acid-binding protein 5 (FABP5): FASN and MAGL generate intracellular fatty-acid pools, whereas FABP5 transports fatty acids to nuclear receptors and promotes pro-metastatic transcriptional signaling. The metastasis-promoting effects of FASN and MAGL were dependent on FABP5 expression in experimental PCa models [45]. These findings identify lipolysis and intracellular lipid trafficking as additional components of PCa metabolic plasticity. However, MAGL expression and activity have not been evaluated according to SARIFA status.

Several of these regulators have also been linked to advanced or treatment-resistant PCa in preclinical studies. CPT1A-dependent fatty-acid oxidation can support tumor growth under androgen-deprived conditions and has been associated with reduced sensitivity to enzalutamide [46]. Conversely, genetic or pharmacological AMPK activation induced a catabolic state characterized by reduced lipogenic potential, proliferation, and invasiveness in experimental PCa models, indicating that the biological effects of AMPK depend on pathway context and cellular state [47]. More recently, combined inhibition of CD36-mediated fatty-acid uptake and SCD1-dependent lipid desaturation suppressed tumor growth, invasion, and metastasis in preclinical models of refractory PCa [48]. Collectively, these findings indicate that PCa progression and therapeutic resistance can be supported by metabolic flexibility across lipid uptake, synthesis, desaturation, and oxidation rather than by a single uniformly dominant pathway. However, CPT1A, SCD1, PPARs, and AMPK signaling have not been investigated according to SARIFA status.

FASN has consequently emerged as a potential therapeutic target in advanced PCa. Early pharmacological inhibitors, including cerulenin and C75, suppressed fatty-acid synthesis and tumor-cell survival in experimental prostate cancer models but had limited suitability for clinical development because of pharmacological and toxicity-related constraints. More selective compounds have subsequently been investigated. The FASN inhibitor IPI-9119 suppressed the expression and transcriptional activity of both full-length AR and AR-V7, inhibited the growth of CRPC xenografts and patient-derived mCRPC organoids, and enhanced the effects of enzalutamide in preclinical models [49]. TVB-2640, also known as denifanstat, inhibited PCa-cell proliferation and altered mitochondrial respiration in experimental models, although the magnitude of the response depended on tumor context [50].

TVB-2640 has demonstrated pharmacodynamic inhibition of FASN and de novo lipogenesis in a first-in-human study of patients with advanced solid tumors, although that study did not establish PCa-specific efficacy [51]. The combination of TVB-2640 and enzalutamide is currently undergoing phase I evaluation in men with metastatic castration-resistant PCa [52]. Thus, pharmacological FASN inhibition has progressed to early clinical investigation in PCa, but its therapeutic efficacy remains unproven. Moreover, no study has evaluated whether SARIFA status, CD36 expression at the invasion front, or relative dependence on exogenous versus de novo synthesized fatty acids predicts sensitivity to FASN inhibition.

**Figure 1:**
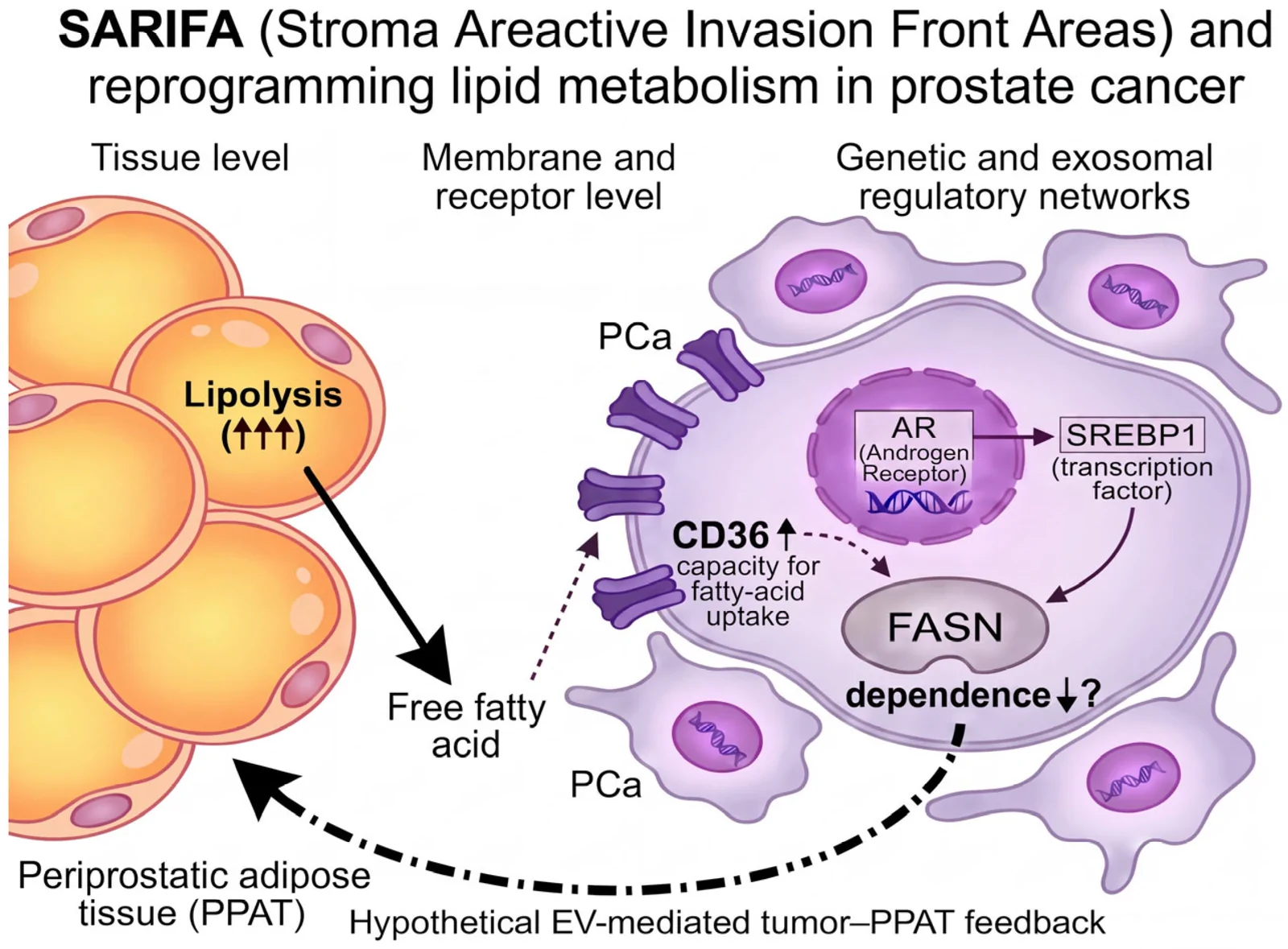
Proposed model of lipid metabolic remodeling at the SARIFA-positive tumor–adipose interface. Lipid metabolism is regulated via androgen receptor (AR) and SREBP1 signaling. The key hypothesis concerns the potentially altered role of FASN: although FASN is traditionally linked to aggressive PCa, SARIFA-positive zones show increased CD36 expression, suggesting an enhanced capacity for the uptake of exogenous fatty acids. We hypothesize that this may reduce the relative dependence of tumor cells on FASN-mediated de novo fatty-acid synthesis. Solid arrows: experimentally supported relationships in PCa, not necessarily SARIFA-specific.; Dashed arrows: proposed SARIFA-specific relationships requiring experimental validation.

Understanding the potential relationship between FASN expression and the SARIFA phenomenon may contribute to the characterization of lipid metabolic remodeling in PCa. De novo lipogenesis is frequently upregulated in PCa, and FASN catalyzes the synthesis of palmitate, which serves as a precursor for more complex lipids [36]. However, FASN expression has not been directly compared between SARIFA-positive and SARIFA-negative areas. By contrast, CD36, a major fatty-acid transporter, was upregulated specifically in tumor cells at SARIFA-positive invasion fronts, indicating increased capacity for the uptake of exogenous fatty acids [12]. These observations raise the hypothesis that SARIFA-positive zones may rely relatively more on exogenous fatty-acid uptake and potentially less on FASN-mediated de novo synthesis. This proposed relationship requires direct experimental evaluation (Figure 1).

The scientific literature currently lacks data regarding the comparative immunohistochemical assessment of FASN expression in SARIFA-positive versus SARIFA-negative areas within the same tumor. However, such studies are critical for supporting or refuting the proposed hypothesis.

### 4.1. AR–Lipid Metabolic Crosstalk in Treatment Resistance and Lineage Plasticity

More broadly, lipid metabolic reprogramming in PCa supports tumor-cell proliferation, adaptation to metabolic stress, and the development of castration-resistant and metastatic phenotypes [34,35,36]. AR signaling coordinates multiple aspects of lipid metabolism, including SREBP1/FASN-dependent de novo lipogenesis and AR-regulated fatty-acid activation pathways involving ACSM1 and ACSM3 [35,53]. Androgen deprivation initially suppresses ligand-dependent AR activity; however, CRPC frequently retains or restores AR signaling, allowing lipid anabolic programs to persist or re-emerge. In a genetically defined subtype of SPOP-mutated PCa with CHD1 loss, SREBP2-driven cholesterol synthesis was shown to increase intratumoral androgen production, enhance AR activity, and promote castration resistance [54]. This finding illustrates a subtype-specific mechanism and should not be generalized to all CRPC.

Lipid metabolic adaptation may also support treatment resistance without a simple restoration of canonical AR-driven lipogenesis. Experimental studies have identified mitochondrial and peroxisomal fatty-acid oxidation enzymes, including DECR1 and DECR2, as contributors to resistance to AR-directed therapy or progression of advanced PCa [55,56]. Thus, treatment-resistant PCa may use different combinations of restored AR signaling, cholesterol and fatty-acid synthesis, uptake of exogenous lipids, and enhanced lipid catabolism. The relative contribution of these pathways is context-dependent, and none has been investigated according to SARIFA status.

Neuroendocrine differentiation represents a distinct resistance trajectory characterized by lineage plasticity and reduced dependence on canonical AR signaling. Experimental studies indicate that inhibition of the AR pathway can relieve AR-mediated repression of lineage-plasticity regulators and facilitate treatment-induced neuroendocrine differentiation [57,58]. Therefore, neuroendocrine PCa should not be interpreted simply as a continuation of the AR-driven lipogenic state, rather, it may involve a reconfigured metabolic program accompanying the loss of luminal AR identity. At present, no direct evidence links SARIFA to response to androgen deprivation or AR pathway inhibitors, progression to CRPC, or neuroendocrine differentiation.

### 4.2. Potential Relationship Between SARIFA and Ferroptosis

Maintaining fatty-acid homeostasis is important for PCa cells to avoid lipotoxicity and ferroptotic cell death [53,59,60]. To date, no published study has directly linked SARIFA to ferroptosis in PCa. This relationship therefore remains a mechanistic hypothesis requiring experimental validation. The rationale for investigating this association is based on the central role of lipid availability, membrane lipid composition, and lipogenic pathways in ferroptosis regulation [59,60,61], together with the increased CD36 expression observed at SARIFA-positive invasion fronts [12].

In the available PCa cohort, SARIFA positivity was associated with increased CD36 expression in tumor cells at the invasion front, suggesting an enhanced capacity for exogenous fatty-acid uptake [12]. Whether this finding is accompanied by altered membrane lipid composition, lipid peroxidation, or ferroptosis sensitivity remains unknown. We hypothesize that a shift toward exogenous fatty-acid uptake, potentially accompanied by altered dependence on FASN-mediated de novo lipogenesis, may influence ferroptosis susceptibility. However, FASN expression, lipid-peroxidation markers, and ferroptosis-defense mechanisms, including GPX4, have not been compared between SARIFA-positive and SARIFA-negative PCa areas.

Oncogenic activation of PI3K–AKT–mTOR signaling can suppress ferroptosis through SREBP-mediated lipogenesis. In preclinical cell-line models, genetic or pharmacological inhibition of SREBP1 increased ferroptosis sensitivity in PI3K–AKT–mTOR-activated cancer cells [61]. These findings provide a mechanistic context for investigating SARIFA but are not SARIFA-specific.

To evaluate the potential relationship between SARIFA and ferroptosis, further studies should include immunohistochemical or spatial mapping of key ferroptosis-related proteins in SARIFA-positive and SARIFA-negative areas; comparative analysis of membrane lipid composition, lipid peroxidation, and ferroptosis-defense pathways; and experimental coculture models reproducing direct interactions between PCa cells and adipocytes. The effects of adipocyte-derived extracellular vesicles could also be examined, followed by treatment with established ferroptosis inducers, such as erastin or RSL3, and inhibitors, such as ferrostatin-1.

## 5. Periprostatic Adipose Tissue as a Key Component of the Prostate Cancer Microenvironment

The periprostatic adipose tissue (PPAT), a key component of the prostate gland microenvironment, participates in the metabolic interplay with cancer cells [34,62]. At the locally invasive front of PCa, direct contact between tumor cells and PPAT represents the defining morphological feature of SARIFA. Although tumor–adipose interactions may contribute to PCa progression, a causal role of the SARIFA phenotype itself has not been established. SARIFA positivity may identify a tumor–adipose interface characterized by altered fatty-acid handling, as supported by the increased CD36 expression observed in tumor cells at SARIFA-positive invasion fronts [12]. Extra-prostatic extension is a common indicator of aggressive disease and an established predictor of poor prognosis in prostate cancer [63]. Direct interactions between prostate cancer cells, adipose tissue, and adipose-derived stem cells play critical roles in promoting tumor extension and progression [64,65,66]. Park et al. described the interplay between prostate cancer cells and stromal adipocytes as pathological feedback loops, mediated through the paracrine secretion of adipokines [67].

PPAT can also be evaluated non-invasively using quantitative MRI. In patients undergoing radical prostatectomy, normalized periprostatic fat volume, calculated as the ratio of periprostatic fat volume to prostate volume, was positively associated with postoperative Gleason grade and pathological stage [68]. In a subsequent retrospective cohort of 162 patients, a higher MRI-measured ratio of periprostatic to subcutaneous adipose tissue thickness was independently associated with shorter time to biochemical recurrence (HR, 1.90; 95% CI, 1.03–3.51; *p* = 0.040 [69]. More recently, MRI radiomic features extracted from periprostatic fat were incorporated with intratumoral radiomic and clinicopathological variables into a model for predicting biochemical recurrence-free survival in 356 patients with non-metastatic PCa after radical prostatectomy [70]. These findings suggest that the amount, distribution, and imaging phenotype of local adipose tissue may contain information related to PCa aggressiveness. However, the available studies are retrospective, use heterogeneous PPAT measurements, and have not evaluated their relationship with histologically defined SARIFA status.

Recent studies have highlighted key mechanisms linking dysfunctional adipose tis-sue to prostate cancer progression. These include the insulin/insulin-like growth factor (IGF) axis, dysregulated adipokine signaling, and the proliferation of stem cell populations in the adipose stromal microenvironment [71,72,73]. However, several studies demonstrated that the adipocyte–cancer cell crosstalk, besides having a direct impact on tumor cells, leads to phenotypical and functional changes in both cell types. Specifically, normal adipocytes are transformed into the so-called “cancer-associated adipocytes” [74]. Following lipolysis induction, mature adipocytes exhibit lipid droplet loss and distinct changes in size and shape that can be effectively visualized using light microscopy. Findings from pancreatic ductal adenocarcinoma suggest that adipocyte lipolysis and the associated morphological changes may facilitate the transfer of lipids, including fatty acids, to adjacent tumor cells [10]. Whether the same process occurs specifically at SARIFA-positive interfaces in PCa remains to be determined.

During cancer progression, epithelial cells can separate from the glandular niche and invade the tumor microenvironment, where exposure to high concentrations of adipokines further promote their proliferation [75,76]. Sacca et al. demonstrated that periprostatic adipose tissue (PPAT) exhibits metabolic dysfunction in patients with PCa. Their findings indicate that adipokines and fatty acids secreted by this tissue act as key drivers of alterations within the tumor microenvironment [17]. The authors suggested that elevated fatty acid levels correlate with prostate cancer aggressiveness, highlighting distinct changes in PPAT metabolism during cancer manifestation and progression. The study also revealed an enrichment of energy production pathways in the PPAT secretome profile of T3-stage tumors relative to T2-stage tumors, suggesting increased energy demands as the disease progresses [17]. Periprostatic adipose tissue plays an essential role in prostate cancer (PCa) aggressiveness. By secreting adipokines, leptin, IL-6, CCL7, FABP4 [77,78], and free fatty acids, PPAT stimulates angiogenesis and promotes tumor growth and metastasis [79]. Furthermore, gene analyses of PPAT samples demonstrated that the expression of genes associated with de novo fatty acid synthesis (e.g., FASN) was significantly lower in PPAT from high-risk PCa than in low-risk counterparts. This was accompanied by the overexpression of inflammatory markers, suggesting that PPAT undergoes functional alterations to meet the high metabolic demands of aggressive tumor cells [79]. These findings are consistent with morphometric observations in SARIFA-positive pancreatic ductal adenocarcinoma, where adipocytes in direct contact with tumor cells showed lipid-droplet loss and reduced cell size [10]. Comparable morphometric evidence is not yet available for SARIFA-positive PCa.

A detailed investigation of adipokines provides accumulating evidence that leptin hyperexpression via the stimulation of STAT3 pathway [80] correlates with a higher Gleason score, increased lymphovascular invasion and reduced survival rates in PCa patients. Recent studies suggest that PPAT-secreted IL-6 drives aggressive prostate cancer phenotype by triggering the epithelial–mesenchymal transition [EMT] [81,82,83]. In this regard, Allott et al. reported a significant association between metabolic syndrome, including obesity, and more aggressive prostate cancer biology and poorer outcomes [84].

The random-effects meta-analysis, assessing the relative risks (RR) of prostate cancer-specific mortality and biochemical recurrence associated with an increase in body mass index (BMI), demonstrated that a 5 kg/m^2^ increase in BMI was associated with 21% higher risk of biochemical recurrence after primary treatment and 15% higher risk of dying of prostate cancer [85].

However, Enke et al. observed no BMI-dependent changes in SARIFA status, suggesting that this SARIFA phenotype is primarily driven by local, rather than systemic, factors [12]. These observations emphasize that BMI and metabolic syndrome represent systemic measures and should not be considered interchangeable with local PPAT volume, distribution, cellular composition, or secretory activity. Obesity may modify PPAT biology and contribute to a permissive tumor microenvironment, but current evidence does not establish obesity or metabolic syndrome as determinants or surrogate markers of SARIFA in PCa. The PPAT serves as a reservoir of adipose-derived mesenchymal stem cells (ADSCs) that can migrate to prostate cancer cells [86]. The role of ADSCs in cancer cell growth warrants further investigation, given their potential bidirectional interactions with the tumor microenvironment. To date, clinical trials have not proven a potential pro-oncogenic role of ADSC enrichment [87]. On the other hand, accumulating results of the in vivo and in vitro preclinical trials demonstrate that ADSCs can promote tumor cell growth in various cancer types [88,89,90]. These findings indicate that ADSCs enhance tumor progression and invasiveness, primarily through the activation of multiple intracellular signaling pathways [91]. It was also demonstrated that ADSCs induce epithelial–mesenchymal transitions (EMT) through paracrine signaling [18]. While investigating EV-mediated communication involving adipose stromal cells, Sacca et al. highlighted that EV-mediated interactions between PCa tumor cells and PPAT are dysregulated in Stage 3 PCa. This disruption implicates cancer pathways involving integrin-mediated cellular communication, EMT, and mRNA stability [17]. Furthermore, this dysregulation may facilitate tumor aggressiveness during extraprostatic expansion. Such interactions may be relevant to the tumor—adipose interface represented by SARIFA; however, direct evidence that PPAT-derived stromal cells or their extracellular vesicles generate the SARIFA phenotype is currently lacking.

## 6. Extracellular Vesicle-Mediated Crosstalk Between PPAT and PCa Cells

Currently, no study has directly linked SARIFA status to extracellular vesicle-mediated communication in PCa. Nevertheless, experimental evidence of bidirectional signaling between PCa cells and adipose tissue provides a biological rationale for investigating this hypothesis. Extracellular vesicles (EVs) are heterogeneous membrane-enclosed particles released by cells through different biogenetic pathways. EVs formed as intraluminal vesicles within multivesicular bodies and subsequently released after fusion with the plasma membrane may be classified as exosomes when their endosomal origin is demonstrated. Vesicles budding directly from the plasma membrane are commonly described as ectosomes or microvesicles. However, according to the MISEV2023 recommendations, operational terms such as small EVs and large EVs are preferable when biogenesis has not been established experimentally [92]. Cross-study comparison remains complicated by differences in EV source material, isolation and purification procedures, size-based classification, molecular characterization, normalization approaches, and functional assays. These methodological variables should be considered when interpreting the biological effects attributed to individual EV populations.

EV cargo may include miRNAs, lncRNAs, circRNAs, mRNAs, proteins, lipids, metabolites, and growth factors. Its composition depends on the cell of origin, cellular state, microenvironmental conditions, and mechanisms of vesicle formation and cargo sorting. Following uptake by recipient cells, EV-associated molecules may modify transcriptional programs, intracellular signaling, metabolism, and cell phenotype [93,94].

EVs may play an important role in the bidirectional interplay between prostate cancer cells and peritumoral adipose tissue. By transporting fatty acids, oxidative enzymes, and metabolites into cancer cells, lipid exosomes act as key drivers of cancer progression [95,96,97,98]. Mature adipocytes secrete several subpopulations of extracellular vesicles (EVs), including small extracellular vesicles (sEVs) and large extracellular vesicles (lEVs). Notably, sEVs are enriched with Alix, the endosomal sorting complexes required for transport I (ESCRT-I) components, and the tetraspanins CD9, CD63, and CD81. In addition, the content of cholesterol is significantly higher in sEVs than in lEVs [99]. Characterizing EVs membrane protein expression offers a promising approach for their potential use in routine clinical practice [100].

In turn, EVs can carry diverse molecular cargo, including mRNAs, miRNAs, lncRNAs, circRNAs, proteins derived from cancer cells transport to the surrounding adipose tissue. These EV-associated cargoes alter the differentiation of adipose cells, driving the generation of tumor-associated adipose tissue and ultimately promoting survival of cancer cells in the microenvironment. Thus, EVs may function as mediators of communication between periprostatic adipose tissue and tumor cells [101]. The microRNA (miRNA) profile of tumor-derived EVs largely mirrors the expression profile of parental tumor cells. Consequently, the EV-mediated transfer of these dysregulated miRNAs can significantly modulate the proliferation and migration of malignant cells [102].

In PCa and other experimental cancer models, EV-mediated communication has been implicated in metabolic reprogramming, angiogenesis, immune modulation, therapy resistance, and the formation of pre-metastatic niches. EV-associated miRNAs, lncRNAs, proteins, and lipids can modify endothelial-cell activation, macrophage polarization, myeloid-cell recruitment, extracellular-matrix remodeling, and the functional state of bone-resident cells. In PCa, EV-associated non-coding RNAs have also been associated with altered AR signaling, epithelial–mesenchymal transition, progression to castration resistance, and remodeling of the bone microenvironment [94,103]. However, these effects depend on the cellular source, molecular cargo, recipient cell type, and disease context. None of these processes has been demonstrated specifically at SARIFA-positive invasion fronts in PCa.

Experimental data indicate that tumor-derived EVs can di-rectly remodel adipocyte metabolism: Lewis lung carcinoma-derived EVs carrying PTHrP, a parathyroid hormone-related protein capable of activating lipolytic signaling in adipocytes, induce adipocyte lipolysis and white adipose tissue browning through the PKA pathway [104]. More directly in prostate cancer, PCa cell-derived EVs were shown to activate the adipocyte lipolytic machinery through suppression of the anti-lipolytic factor G0S2, induction of the lipase ATGL, and activation of the cAMP/PKA/HSL cascade there-by increasing free fatty acid release; these fatty acids subsequently enhanced PCa cell pro-liferation and clonogenicity [19]. Tumor–adipose tissue crosstalk has been reported by Liu et al., who showed that gastric cancer-derived exosomal miR-155 suppresses adipo-genesis and promotes brown adipose differentiation of adipose mesenchymal stem cells via the C/EBPβ pathway, thereby contributing to cancer-associated cachexia [105]. Accord-ing to Fontana et al., EVs derived from 3T3-L1 adipocytes promoted PCa aggressiveness, inducing increased proliferation, invasion, and drug resistance [20].

**Figure 2:**
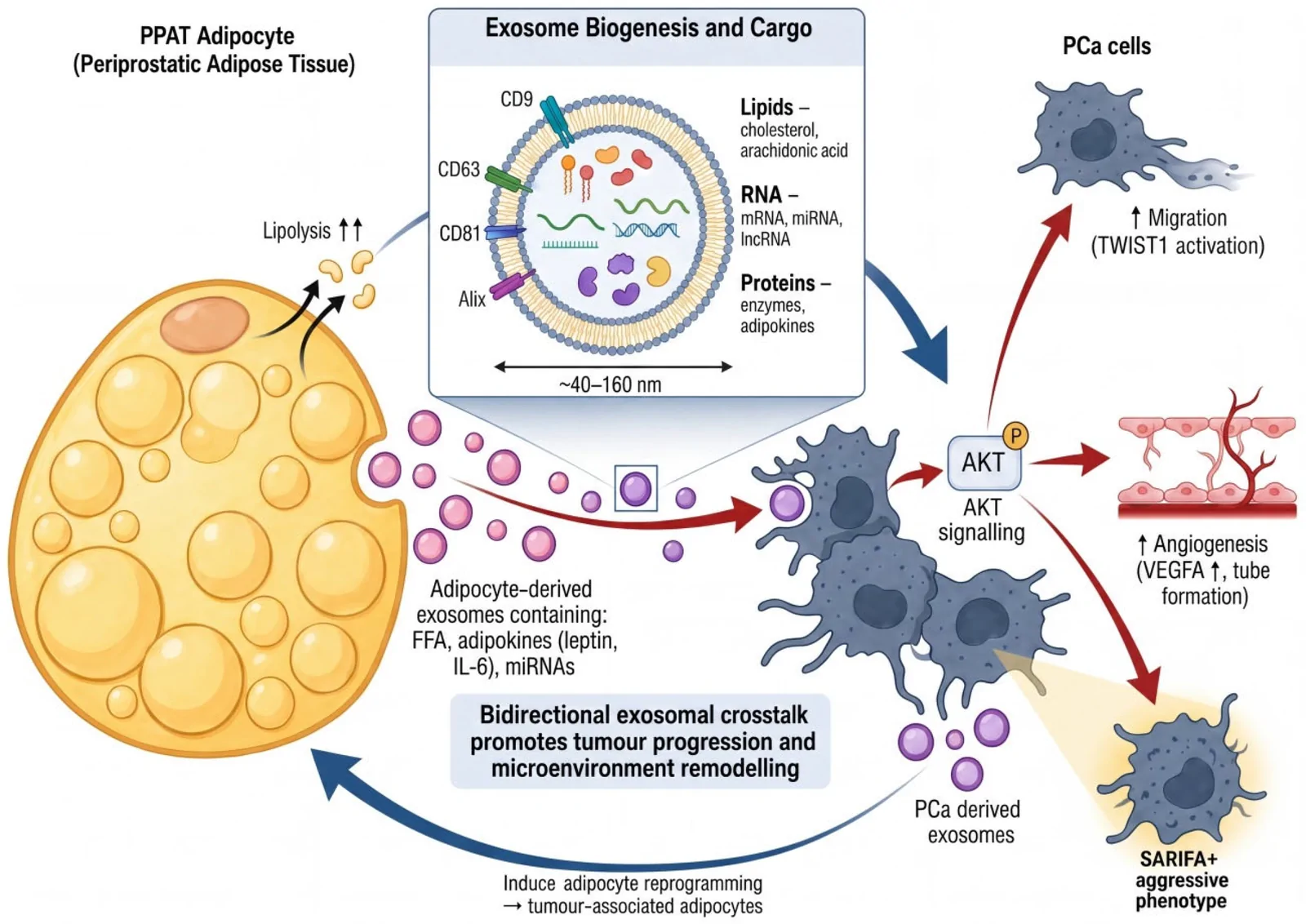
Proposed bidirectional extracellular vesicle-mediated crosstalk between PPAT and PCa cells and its hypothetical relationship with SARIFA. Adipocyte- and PPAT-derived extracellular vesicles can stimulate PCa cell proliferation and migration, activate pro-angiogenic and metabolic signaling, and may contribute to biological processes relevant to the SARIFA-positive tumor–adipose interface. In turn, PCa-derived extracellular vesicles may induce lipolytic and phenotypic changes in adipocytes. A direct role of these interactions in the formation of SARIFA has not yet been demonstrated.

Typically, the production of adipocyte-derived EVs is higher before adipogenesis. At this stage, the EVs are enriched in arachidonic acid and adipogenesis markers, such as PREF-1 and PPARγ, whilst adiponectin content in adipocyte-derived EVs was increased on day 15 of the differentiation of pre-adipogenic cells. Since the uptake of adipocyte-derived EVs promotes the growth of various tumors, including breast cancer, it is suggested that the cargo and lipid composition of the EVs depend on the differentiation stage of the adipo-cytes [106]. Recent research demonstrated that EVs derived from the periprostatic adipose tissue (PPAT) of PCa patients modulate tumor cell behavior in distinct ways, depending on the tumor grade. EVs from low-risk (ISUP I–II) PCa patients primarily stimulated tumor proliferation and induced M2 polarization of macrophages. In contrast, EVs from high-risk (ISUP III–V) PCa patients enhanced tumor cell migration via TWIST1 upregulation and exerted pro-angiogenic effects. Consistent with the observed angiogenic effects, VEGFA gene expression was significantly increased. Notably, both low- and high-PPAT EVs activated the AKT signaling pathway which serves as a central regulator of PCa cell survival and metabolic adaptation. These findings suggest that PPAT-derived extracellular vesicles can modulate several processes relevant to PCa progression, including proliferation, migration, angiogenesis, macrophage polarization, and AKT signaling [21]. Because SARIFA represents direct tumor–adipose contact, extracellular vesicle-mediated signaling is a plausible but currently unproven contributor to this phenotype (Figure 2).

Further studies should determine whether the SARIFA-positive tumor–adipose interface is associated with altered EV release, uptake, or molecular cargo compared with SARIFA-negative tumor areas. Spatially resolved analyses of EV-associated RNAs, proteins, lipids, and recipient-cell responses could help identify the direction and functional consequences of communication between PCa cells, adipocytes, stromal cells, and immune cells. Circulating or tissue-associated EV markers may eventually provide surrogate information concerning this interface; however, their ability to identify SARIFA status or predict its clinical significance has not yet been evaluated.

Although several of the cited studies provide PCa-specific evidence for bidirectional communication between tumor cells and adipose tissue [17,19,20,21], other mechanistic observations were derived from non-prostate cancer or general adipocyte models [93,95,96,97,98,99,100,101,102,104,105,106]. None of these studies compared EV release, uptake, molecular cargo, or functional effects according to SARIFA status. Consequently, EV-mediated crosstalk represents a biologically plausible mechanism relevant to the tumor–adipose interface, but it has not been established as a defining component or causal determinant of SARIFA in PCa.

## 7. The Immune Microenvironment and Its Potential Relationship with SARIFA

Recently, research interest has been focused on the possible link between the regulation of lipid metabolism and immune surveillance. It remains unclear how tumor-associated lipid metabolism in prostate cancer regulates immune responses and whether the altered immune profiles drive the aggressive phenotype seen in SARIFA-positive tumors.

Independent of SARIFA status, PCa is generally characterized by a relatively immunosuppressive and weakly inflamed tumor microenvironment. Single-cell analysis of localized PCa demonstrated that high-grade tumors were enriched in exhausted CD8+ T cells, regulatory T cells, and myeloid populations, whereas lower-grade tumors showed a higher relative abundance of activated CD8+ effector cells [107]. Immunosuppressive myeloid populations in PCa include tumor-associated macrophages and myeloid-derived suppressor cells, which can inhibit T-cell activation through cytokine production, metabolic competition, and immune-checkpoint pathways. Dendritic-cell dysfunction may further impair antigen presentation and T-cell priming. Tumor-associated neutrophils can also adopt heterogeneous functional states, and their contribution may vary according to tumor stage and microenvironmental context [108,109].

Consistent with these findings, exhausted CD8+ T cells in localized high-grade PCa express inhibitory receptors including PD-1, CTLA-4, TIM-3, and TIGIT. These immune states may contribute to the generally limited activity of immune-checkpoint blockade in unselected advanced PCa, although selected molecular subgroups may derive greater benefit [110]. To our knowledge, neither immune-checkpoint expression nor the spatial distribution of regulatory T cells, myeloid-derived suppressor cells, dendritic cells, or tumor-associated neutrophils has been evaluated specifically at SARIFA-positive invasion fronts in PCa [12,24].

In a large colorectal cancer (CRC) cohort (N = 1876), Tapiainen et al. demonstrated that SARIFA positivity was associated with lower densities of CD3+ T cells, macrophages with an M1-like phenotype, and CD66b+ granulocytes, together with higher densities of macrophages with an M2-like phenotype [111]. This pattern was interpreted as compatible with a more immunosuppressive local microenvironment.

In the study by Grosser et al., tissue cytokine expression was assessed by RNA in situ hybridization for cytokines expression analysis combined with CD68 immunohistochemistry in gastric carcinoma and gastroesophageal junction adenocarcinoma sections. IL-10 and IL-12 showed no significant SARIFA-dependent differences, whereas IL-6 and TNF-α expression was significantly lower at the invasion front of SARIFA-positive tumors [112]. A marked reduction in natural killer (NK) cells was observed in the peripheral blood of SARIFA-positive CRC patients. In addition, immunohistochemical evaluations indicated a notable decrease in NK cells and NK-like lymphocytes within the SARIFAs of SARIFA-positive tumors [23].

Taken together, studies in colorectal and gastric cancers associate SARIFA positivity with alterations in local and systemic immune profiles [23,111,112]. These findings support further investigation of the immune context of SARIFA but cannot be directly extrapolated to PCa. At present, there is no direct evidence that SARIFA-positive PCa is characterized by a distinct immune-cell composition, cytokine network, immune-checkpoint profile, or mechanism of immune evasion. Future studies should compare SARIFA-positive and SARIFA-negative tumor–adipose interfaces using spatially resolved and multiplex approaches. Relevant analyses should include macrophage states, tumor-associated neutrophils, regulatory and cytotoxic T cells, myeloid-derived suppressor cells, dendritic cells, NK cells, immune-checkpoint molecules, and cytokine–chemokine networks. Such studies should determine whether immune alterations are localized specifically to the SARIFA interface or reflect the broader immune phenotype of the tumor.

The absence of SARIFA-specific immune data is also relevant to the interpretation of its potential relationship with immunotherapy. At present, there is no evidence that SARIFA identifies an immune phenotype associated with response or resistance to immune-checkpoint inhibition, and SARIFA should not be regarded as an immunotherapy biomarker in PCa.

The direct evidence supporting different components of the proposed SARIFA-associated phenotype varies substantially among cancer types. To distinguish demonstrated findings from relationships inferred for PCa, the available evidence is summarized in Table 2.

## 8. An Integrative Model of the SARIFA-Associated Tumor–Adipose Interface in PCa

The available evidence supports a multistep model in which intrinsic metabolic remodeling of PCa cells and signals derived from periprostatic adipose tissue converge at the tumor invasion front. Androgen receptor and SREBP-dependent pathways promote lipid synthesis and metabolic plasticity in PCa cells [16,34,35]. Independently, PPAT can provide fatty acids, adipokines, and extracellular vesicles that modulate tumor-cell proliferation, migration, angiogenic signaling, and metabolic adaptation [19,20,21,26,79]. These processes have been demonstrated in PCa models, but most were not examined in relation to SARIFA status.

Direct SARIFA-specific evidence in PCa is currently limited to the association of SARIFA positivity with adverse pathological characteristics and increased CD36 expression in tumor cells at the invasion front [12]. Increased CD36 expression suggests an enhanced capacity for exogenous fatty-acid uptake, but direct measurements of lipid transfer, fatty-acid oxidation, or reduced dependence on de novo lipogenesis in SARIFA-positive areas have not been performed. The proposed shift from FASN-dependent lipid synthesis toward the utilization of adipocyte-derived fatty acids therefore remains a mechanistic hypothesis.

Bidirectional signaling between tumor cells and adipose tissue may reinforce this interface. PCa-derived extracellular vesicles can stimulate adipocyte lipolysis, whereas PPAT- and adipocyte-derived extracellular vesicles can promote several tumor-associated phenotypes [19,20,21]. However, their direct contribution to SARIFA formation has not been demonstrated. Similarly, immune alterations associated with SARIFA have been reported in colorectal and gastric cancers [23,111,112], but comparable evidence is not yet available in PCa. Thus, SARIFA may represent the morphological endpoint of coordinated metabolic and microenvironmental interactions, although the causal sequence and relative contribution of each component require experimental validation.

### Experimental Strategies for Mechanistic Validation of SARIFA

Because SARIFA is defined by a spatially restricted tumor–adipose interface, bulk profiling of whole tumor specimens may dilute or obscure molecular signals specific to this region. Future studies should therefore prospectively annotate SARIFA-positive and SARIFA-negative areas within the same radical prostatectomy specimens and compare them using spatially resolved approaches. Spatial transcriptomics combined with single-cell or single-nucleus RNA sequencing could identify tumor, adipocyte, stromal, endothelial, and immune-cell states and reconstruct ligand–receptor interactions at the invasive front. Multiplex imaging and spatial proteomics could determine whether the SARIFA interface is characterized by specific cellular neighborhoods and spatial expression patterns involving AR, SREBP1, FASN, CD36, FABP4, CPT1A, SCD1, ferroptosis-related proteins, immune-checkpoint molecules, and macrophage or lymphocyte states. Recent studies have demonstrated the feasibility of integrating single-cell and spatial transcriptomic analysis in PCa and of mapping spatial stromal and immune-cell organization by multiplex imaging, although these approaches have not yet been applied specifically to SARIFA [114,115].

Comparative lipidomic and metabolomic analyses should complement spatial transcriptomic and proteomic profiling. Laser-capture microdissection or other region-resolved sampling of SARIFA-positive and SARIFA-negative interfaces could be used to quantify free fatty acids, phospholipids, neutral lipids, acylcarnitines, cholesterol-related metabolites, lipid-peroxidation products, and other metabolic intermediates. Such analyses could test whether increased CD36 expression at SARIFA-positive fronts is accompanied by altered lipid uptake, fatty-acid oxidation, membrane lipid composition, or reduced relative dependence on de novo lipogenesis. Integrated spatial proteogenomic approaches combining morphology, spatial gene expression, protein localization, and genomic or epigenomic data may further distinguish tumor-intrinsic alterations from signals derived from adjacent adipocytes, stromal cells, or immune cells. A recent spatial multi-omics study in PCa combined spatial transcriptomics with bulk transcriptomics, proteomics, DNA methylation analysis, and tissue staining, illustrating the feasibility of such integrative designs [116].

Functional validation requires experimental systems that reproduce direct or near-direct interactions between PCa cells and adipocytes. Patient-derived prostate cancer organoids can preserve selected histological and genomic characteristics of the parental tumor and permit genetic manipulation and pharmacological testing. However, epithelial organoids alone do not reproduce the native adipose, stromal, vascular, or immune compartments of the SARIFA interface. SARIFA-oriented models should therefore combine patient-derived organoids or prostate cancer cells with primary PPAT-derived adipocytes, adipose stromal cells, fibroblasts, and, where appropriate, immune cells. Direct-contact and transwell configurations should be compared to distinguish contact-dependent effects from soluble-factor- or EV-mediated communication. Relevant functional readouts should include adipocyte delipidation, fatty-acid transfer, tumor-cell invasion, CD36 and FASN expression, fatty-acid oxidation, cytokine and EV release, ferroptosis sensitivity, and response to androgen-receptor pathway inhibition [117,118].

Microfluidic organ-on-chip systems could provide additional control over spatial organization, extracellular-matrix composition, nutrient and oxygen gradients, fluid flow, and the exchange of soluble factors and EVs between tumor and adipose compartments. Separate but communicating channels would permit comparison of paracrine signaling with direct tumor–adipocyte contact, while live imaging could be used to follow invasion and adipocyte remodeling over time. Prostate-cancer-on-chip platforms have been developed, but no organ-on-chip model currently reproduces or validates the SARIFA morphology [119].

Genetically engineered mouse models may complement ex vivo systems by allowing tumor progression to be studied in an immunocompetent organism. Models based on prostate-specific Pten loss, with additional Trp53 and/or Rb1 alterations, reproduce selected features of invasive disease, castration resistance, metastasis, or lineage plasticity. They could be used to determine whether spontaneous invasion into periprostatic adipose tissue generates a stroma-areactive interface resembling human SARIFA and whether this morphology changes after androgen deprivation or metabolic intervention. However, species-specific differences in prostate anatomy, adipose distribution, tumor histology, and immune composition require direct morphological comparison with human prostatectomy specimens. Consequently, demonstration of tumor–adipocyte contact in a mouse model should not automatically be classified as SARIFA without applying predefined histological criteria [120,121].

A rigorous experimental program should therefore combine spatial profiling of human SARIFA-positive tissue with reductionist tumor–adipocyte models and in vivo validation. Concordance across morphology, molecular signatures, functional assays, and animal models would be required before a causal mechanism of SARIFA formation could be proposed.

## 9. SARIFA and Artificial Intelligence Technologies

The advancement of artificial intelligence (AI) and deep learning (DL) algorithms is opening new prospects for the automated analysis of histopathological slides and the identification of prognostic biomarkers. The SARIFA phenomenon serves as a unique example of how independently developed approaches—traditional human expertise in evaluating tissue samples and machine learning algorithms—can identify meaningful morphological features. Together, these methods provide a platform for convergence and mutual enrichment [113].

Independently of the SARIFA concept, the Tumor–Adipose Feature (TAF) was identified through post hoc interpretation of a weakly supervised deep-learning system trained to predict disease-specific survival from unannotated H&E-stained whole-slide images of stage II and III CRC. To generate human-interpretable histological features, embeddings obtained from tumor-containing image patches were grouped using unsupervised clustering. The cluster most strongly associated with high model-predicted risk was characterized by small, moderately to poorly differentiated tumor-cell clusters adjacent to adipose tissue and was subsequently termed TAF [122]. TAF was therefore not defined a priori by pathologists but emerged as a model-derived image pattern that was subsequently assigned a histomorphological interpretation. External pathologist-based evaluation later demonstrated that TAF could be scored semiquantitatively as absent, unifocal, multifocal, or widespread in an independent colon cancer cohort [123].

TAF and SARIFA show substantial morphological overlap but should not be considered equivalent. In a pathological review of 175 published image patches classified computationally as TAF, 117 also fulfilled the histological criteria for SARIFA [113]. TAF is principally characterized by the proximity of tumor cells to adipocytes and may include intervening inflammatory or stromal tissue, whereas SARIFA requires direct tumor-cell–adipocyte contact without an intervening desmoplastic or inflammatory stromal response. Accordingly, TAF was also identified in 45 of 138 SARIFA-negative CRC cases. Bulk RNA-sequencing analyses further indicated stronger increases in CD36 and FABP4 expression in SARIFA-positive/TAF-positive cases than in SARIFA-negative/TAF-positive cases [113]. These observations suggest that direct tumor–adipocyte contact may define a more specific biological phenotype than tumor–adipose proximity alone.

The interpretability of TAF nevertheless has important limitations. TAF represents a post hoc explanation of a prognostic model rather than a prospectively predefined histological variable, and its identification may depend on the training population, slide preparation and scanning, tumor-region selection, image-patch sampling, embedding model, and clustering procedure. Recognition of a model-derived pattern by pathologists does not demonstrate that this feature is the sole determinant of model predictions or that it has a causal relationship with clinical outcome. Moreover, the TAF–SARIFA comparison and its molecular associations have been evaluated in CRC rather than PCa. TAF should therefore be regarded as a partially interpretable, model-derived feature with morphological overlap with SARIFA, not as an automated surrogate for SARIFA in PCa.

Foersch et al. developed a deep-learning algorithm to predict prognosis and therapy response using an immune score-based multilayer approach. The generation of images with this algorithm revealed an adipocytes-close-to-tumor-cells feature as an immune reaction—an independent pattern that was prognostically relevant [124]. Jiang et al. came to similar conclusions in their multicenter colorectal cancer (CRC) prognosis study, high-lighting the importance of tumor-infiltrated fat/tumor–adipocyte interaction for poor prognosis [125].

Overall, deep-learning algorithms may support more standardized detection and quantification of tumor–adipose features in large patient cohorts. However, the available evidence is predominantly derived from colorectal cancer, and the reproducibility and clinical performance of automated SARIFA assessment in PCa remain to be established.

### Future Directions for Multimodal AI and Precision Oncology

Future computational research should move beyond the automated identification of SARIFA as a binary histomorphological feature. Multimodal models could integrate quantitative characteristics of the tumor–adipose interface with serum PSA, Grade Group, pathological stage, MRI- or PSMA-PET/CT-derived features, tissue genomic classifiers, spatial molecular profiles, longitudinal PSA kinetics, and treatment data. Depending on the study design, such models could be developed to estimate biochemical recurrence, metastasis-free survival, prostate cancer-specific survival, or treatment benefit. Digital pathology-based multimodal artificial intelligence models integrating histological images with clinical variables have already shown prognostic associations in independent PCa cohorts and have been evaluated using material from randomized clinical trials [126,127]. However, these findings cannot be extrapolated to SARIFA, and no published model has demonstrated that the inclusion of SARIFA provides incremental prognostic or predictive information beyond established variables.

Radiopathomics may provide a framework for linking preoperative imaging phenotypes to matched histopathological features. In this context, radiopathomics refers to the spatial or computational integration of radiological images with digitized tissue morphology, rather than to the prediction of tumor genomic characteristics from imaging. For SARIFA, patient-specific registration of MRI or PSMA-PET/CT with whole-mount prostatectomy sections could be used to determine whether SARIFA-positive tumor–adipose interfaces have reproducible imaging correlates. Deep-learning pipelines have demonstrated the technical feasibility of registering prostate MRI with whole-mount histopathology [128]. However, no imaging signature of SARIFA has been identified or externally validated, and the spatial resolution of clinical imaging may be insufficient to visualize direct tumor-cell–adipocyte contact itself. Any proposed imaging surrogate would therefore require histopathological confirmation and independent multicenter validation.

Machine-learning models intended for survival prediction should account explicitly for time-to-event outcomes and censoring and should be evaluated using external validation, calibration, and clinically interpretable measures of net benefit. High discrimination alone would not establish clinical utility. Similarly, a model associated with outcome should not be described as treatment-predictive unless treatment effects differ between model-defined groups and a treatment-by-model interaction is demonstrated. For SARIFA, AI-assisted personalized decision-making should therefore remain a future objective contingent on analytical validation of SARIFA assessment, demonstration of incremental prognostic value, and prospective evaluation of whether the model changes management or improves outcomes [129].

A digital twin represents a more distant and conceptually distinct application. In precision oncology, the term denotes a patient-specific computational model that is updated over time using longitudinal clinical, imaging, pathological, molecular, and treatment-response data. It should not be used as a synonym for a static prognostic score or a single AI classifier. A future SARIFA-oriented digital twin could theoretically integrate tumor–adipose morphology, imaging, molecular profiles, PSA trajectories, and treatment exposures to simulate alternative disease courses or therapeutic strategies. However, digital-twin research in PCa remains at an early stage, and no clinically validated PCa digital twin currently incorporates or predicts SARIFA status [130]. Digital twins should therefore be presented as a long-term research concept rather than as an immediately applicable clinical technology.

Accordingly, the immediate computational priority is not the construction of a SARIFA-based digital twin, but the development and external validation of transparent models that can reproducibly identify SARIFA, quantify its extent, and determine whether it adds clinically meaningful information to existing risk-stratification approaches.

## 10. Requirements for Validation, Reporting Standardization, and Potential Clinical Implementation

Before SARIFA can be considered for clinical use in PCa, its analytical validity, clinical validity, and clinical utility should be evaluated separately. Analytical validation requires standardized definitions of the tumor—adipose interface, the minimum amount of evaluable tissue, the unit of assessment, and the threshold used to define SARIFA positivity. The reproducibility of binary and quantity-dependent scoring should be examined in blinded intraobserver and interobserver studies across different institutions. These studies should also determine whether results are consistent between whole-mount large-format sections and routinely sampled conventional prostatectomy blocks and should account for pre-analytical variables such as fixation, block selection, sectioning, and H&E staining quality.

Standardization of pathology reporting would require a consensus-based scoring and reporting framework. Such a framework should specify the eligible specimen type, minimum extent of the evaluable tumor–adipose interface, number and type of sections to be examined, unit of assessment, binary or quantitative scoring approach, positivity threshold, and procedures for resolving equivocal cases. It should also define how multifocal tumors, heterogeneous SARIFA distribution, cautery or tissue-processing artifacts, and cases lacking an adequately sampled tumor–adipose boundary should be reported. Current prostatectomy reporting frameworks illustrate the level of standardization required for routine implementation and recommend structured or synoptic reporting of validated pathological variables [14,131]. Before inclusion in such reporting frameworks could be considered, SARIFA should first be evaluated as a clearly defined research variable in independent prospective studies.

Clinical validity should be assessed in independent multicenter cohorts with adequate numbers of clinically relevant events. Such studies should determine whether SARIFA provides prognostic information beyond established variables, including serum PSA, Grade Group, pathological tumor and lymph node stage, extraprostatic extension, and surgical margin status [5,12]. Evaluation should include multivariable models, discrimination and calibration measures, and assessment of the incremental prognostic value of SARIFA rather than relying solely on univariable associations. Future prognostic studies should also be reported in accordance with the REMARK recommendations, including transparent descriptions of patient selection, specimen handling, marker assessment, clinical endpoints, statistical methods, and effect estimates [132]. Automated or artificial intelligence-assisted assessment would require separate external validation across scanners, laboratories, and patient populations.

Only after analytical and clinical validation should the contribution of SARIFA to established postoperative risk-stratification models be evaluated. Its addition to models based on PSA, Grade Group, pathological stage, lymph node status, extraprostatic extension, and surgical margin status should be tested using prespecified model-updating procedures in independent cohorts. Evaluation should include discrimination, calibration, decision-curve analysis, and assessment of whether SARIFA provides clinically meaningful net benefit or changes risk classification beyond established variables. Model development and validation datasets should be separated, and scoring thresholds should not be selected and evaluated in the same cohort. Until such analyses demonstrate incremental clinical value, SARIFA should not be incorporated into treatment-selection algorithms.

Regulatory requirements would depend on the intended context of use and the method of assessment. A pathologist-assessed H&E feature intended for postoperative prognostic stratification, an automated image-analysis system, and a biomarker proposed for treatment selection represent different applications and may require different evidence and regulatory pathways. The FDA–NIH BEST framework distinguishes analytical validation of the measurement method from clinical validation of the biomarker for its proposed use [133]. Formal biomarker qualification is also context-specific and does not itself constitute approval of a diagnostic test or software device [134]. Because the current evidence does not establish independent prognostic or predictive utility in PCa, consideration of formal regulatory acceptance would be premature.

To clarify its distinct informational domain and current level of evidence, SARIFA should be evaluated as a potential adjunct to, rather than a replacement for, established clinicopathological features and emerging molecular, circulating, imaging, and computational biomarkers (Table 3).

Clinical utility and cost-effectiveness should be evaluated only after analytical and clinical validity have been demonstrated. The possibility of assessing SARIFA on routinely prepared H&E-stained sections eliminates the need for an additional stain or molecular assay, but does not by itself establish cost-effectiveness. Pathologist time, training, the number of sections requiring review, digital pathology infrastructure, and potential confirmatory procedures should also be considered. Ultimately, prospective studies would be required to determine whether the addition of SARIFA to existing risk-stratification approaches changes clinical decisions or improves patient outcomes.

## 11. Conclusions

SARIFA is a recently described histomorphological feature associated with unfavorable clinicopathological characteristics in a single retrospective PCa cohort; however, the available evidence does not establish its independent prognostic value [12]. Metastasis-free survival and treatment response have not been evaluated according to SARIFA status, and there is currently no evidence supporting its use as a predictive marker for androgen-directed therapy, radiotherapy, chemotherapy, or immunotherapy. A major limitation of SARIFA assessment is the requirement for a surgical specimen containing an evaluable tumor–adipose interface. Therefore, the identification of validated surrogate markers remains an important research objective.

The interaction with periprostatic adipose tissue at the invasive front, morphologically represented by SARIFA, may be an important feature of locally advanced prostate cancer. SARIFA positivity may reflect tumor metabolic remodeling involving an altered balance between de novo lipid synthesis and the uptake of exogenous fatty acids. However, the causal relationship between these metabolic processes and the formation of the SARIFA phenotype has not yet been demonstrated directly.

Future investigations should focus on the EV-mediated mechanisms and transcriptional regulators that may mediate communication between tumor cells, adipose tissue, and immune cells in the SARIFA-associated phenotype. Further characterization of SARIFA-associated molecular mechanisms, including its potential relationship with ferroptosis, may improve understanding of the biological significance of this morphology. Future studies should prioritize standardized assessment criteria, intra- and interobserver reproducibility, prospective external validation, and evaluation of the incremental prognostic value of SARIFA before its potential clinical utility can be determined.

## Figures and Tables

**Table 1:** Published primary evidence on SARIFA in prostate cancer.

Study and Cohort	SARIFA Assessment	Main Findings	Main Limitations
Enke et al., 2024 [12]; retrospective single-center cohort of 301 patients with pT3a PCa treated by radical prostatectomy; whole-mount sections	Binary assessment based on ≥1 SARIFA-positive area; additional quantity-dependent classification based on positivity in ≥1/3 of examined slides	SARIFA positivity was associated with positive surgical margins, non-focal extraprostatic extension, higher Gleason scores/Grade Groups, and a higher number of positive lymph nodes among pN1 cases. CD36 expression was increased at SARIFA-positive invasion fronts, whereas FABP4 did not differ. Associations with overall survival and biochemical recurrence-free survival were not statistically significant; PSMA-PET/CT subgroup findings were exploratory and non-significant	Restricted to pT3a prostatectomy cases; no independent validation cohort; small outcome subgroups; cohort-derived quantitative threshold; no formal PCa-specific reproducibility study; biopsy feasibility, incremental prognostic value, predictive utility, and clinical utility remain unvalidated

Note: No independent primary PCa cohort validating these findings has been published to date. Review articles were not included because they do not report additional original PCa data.

**Table 2:** SARIFA evidence by cancer type: directly demonstrated findings and inferences for prostate cancer.

Cancer Type	Directly Demonstrated SARIFA Findings	Relevance and Limitations for PCa
Prostate cancer	One retrospective single-center cohort of 301 patients with pT3a PCa showed associations of SARIFA positivity with adverse pathological characteristics and increased CD36 expression in tumor cells at the invasion front [12]. Associations with overall survival and biochemical recurrence-free survival were not statistically significant	This is the only direct PCa-specific evidence. SARIFA-associated immune alterations, extracellular vesicle signaling, lipid transfer, ferroptosis susceptibility, and treatment response have not been demonstrated in PCa
Colorectal cancer	Multiple cohorts have associated SARIFA with adverse clinical outcomes, stromal morphometry, altered local and systemic immune profiles, changes in NK cells, and differences in macrophage, T-cell, and granulocyte densities [8,9,22,23,111]. Computational tumor–adipose features partly overlap with histological SARIFA [113]	These findings support investigation of immune and stromal mechanisms in PCa but cannot establish that the same cellular composition, prognostic effect, or computational phenotype occurs at the prostate tumor–adipose interface
Gastric and gastroesophageal junction cancers	SARIFA has been associated with adverse outcomes, tumor-promoting adipocyte interactions, and altered cytokine expression at the invasion front, including lower IL-6 and TNF-α expression in SARIFA-positive tumors [6,112]	These data provide indirect support for tumor–adipose and immune interactions but do not demonstrate comparable cytokine or immune alterations in SARIFA-positive PCa
Pancreatic ductal adenocarcinoma	SARIFA positivity has been associated with adverse clinical characteristics and outcomes, supporting the applicability of the tumor–adipose interface concept in another lipid-rich tumor microenvironment [10]	Adipocyte lipolysis and lipid transfer may be mechanistically relevant to PCa, but their direct contribution to SARIFA formation in PCa has not been established
Oesophagogastric cancer	SARIFA improved prognostic risk stratification in patients receiving perioperative chemotherapy in analyses of the MAGIC and ST03 trial cohorts [11]	This finding does not demonstrate treatment-predictive utility in PCa. No treatment-by-SARIFA interaction has been evaluated for androgen-directed therapy, radiotherapy, chemotherapy, or immunotherapy in PCa

Note: Findings obtained in non-prostate tumors are presented as indirect biological or methodological evidence. They should not be interpreted as validation of SARIFA-associated mechanisms, prognostic value, or treatment-predictive utility in PCa.

**Table 3:** Positioning of SARIFA relative to established and emerging prognostic features and biomarker classes in prostate cancer.

Assessment and Required Material	Principal Biological or Pathological Information	Current Prognostic or Clinical Position	Main Advantages	Main Limitations
Established clinicopathological assessment: serum PSA; Gleason score and ISUP Grade Group; pathological stage; lymph node status; extraprostatic extension; lymphovascular and perineural invasion; surgical margin status (blood, biopsy, and radical prostatectomy material, as applicable)	Tumor burden, architectural differentiation, anatomical extent, and routes of local or systemic spread	Standard basis for clinical and postoperative risk stratification. Grade Group, pathological stage, lymph node status, extraprostatic extension, lymphovascular invasion, and margin status provide established prognostic information; the independent value of perineural invasion is less consistent [5,14]	Widely available; standardized; incorporated into clinical risk models and routine pathology reporting	Sampling limitations and interobserver variability; several parameters are available only after surgery; do not directly characterize the tumor–adipose interface
Adverse histomorphological patterns: invasive cribriform carcinoma, intraductal carcinoma, and tumor budding (H&E-stained biopsy or prostatectomy tissue)	Adverse glandular or intraductal architecture and invasive phenotype	Invasive cribriform carcinoma and intraductal carcinoma are associated with adverse outcome and are recommended for reporting. Tumor budding remains an emerging, less standardized feature [5,13,14]	Assessable on routine histology; provides architectural information complementary to Grade Group	Definitions, sampling, and interobserver reproducibility remain relevant limitations; tumor budding is not routinely standardized in PCa; these features do not specifically assess tumor–adipocyte interaction
Tissue-based genomic classifiers: Decipher, Prolaris, Oncotype DX Genomic Prostate Score, and ProMark (biopsy or prostatectomy tissue, depending on the assay)	Tumor-intrinsic gene-expression or protein programs associated with biological aggressiveness	Selective adjunct when the result is likely to affect management; routine use in all patients is not recommended [14,15]	Provides molecular information complementary to conventional clinicopathological variables	Requires dedicated testing; assay-specific indications and validation; additional cost; prospective evidence that routine use improves long-term outcomes remains limited
Circulating biomarkers: circulating tumor DNA, circulating tumor cells, and extracellular vesicle-associated biomarkers (blood or plasma)	Tumor-derived genomic, cellular, or vesicular material potentially reflecting tumor burden, clonal evolution, dissemination, and systemic signaling	Investigational for risk stratification in localized PCa. Ultrasensitive ctDNA detection has shown potential association with recurrence risk, whereas CTC detection has limited sensitivity in localized disease [135,136]	Minimally invasive; potentially repeatable and suitable for longitudinal monitoring	Low tumor-derived signal in localized disease; assay and pre-analytical heterogeneity; clinical thresholds are not standardized; no validated relationship with SARIFA status
Radiomic and radiogenomic biomarkers: quantitative MRI- or PSMA-PET/CT-derived features, alone or integrated with tumor genomic or transcriptomic data	Quantitative whole-lesion imaging phenotype and spatial heterogeneity; radiogenomic models additionally evaluate associations between imaging features and molecular characteristics of the tumor.	Investigational. Some radiomics models have shown external prognostic validation, but routine clinical integration remains unestablished (radiomics: [137,138,139]; radiogenomics: [140,141]	Non-invasive; evaluates the entire visible lesion and potentially multifocal disease; may be assessed before treatment	Dependence on image acquisition, segmentation, feature extraction, and model transportability; requires independent multicenter validation; no validated radiological surrogate for SARIFA
Artificial intelligence-assisted pathology biomarkers: computational features derived from digitized H&E slides	Quantitative image-derived morphology, including spatial tumor–stroma and tumor–adipose relationships	Investigational. Tumor–adipose features identified by deep learning overlap partly with SARIFA in colorectal cancer, whereas automated SARIFA assessment has not been validated in PCa [113,122,123,124,125,142]	Scalable and quantitative; may reduce observer-dependent variability and analyze large tissue areas	Scanner and laboratory domain effects; requirements for annotation, external validation, interpretability, and digital infrastructure; current tumor–adipose evidence is predominantly derived from colorectal cancer
SARIFA: H&E-stained radical prostatectomy sections containing an evaluable tumor–adipose interface	Direct contact between tumor cells and adipocytes without intervening desmoplastic or inflammatory stroma; candidate morphological correlate of local tumor–adipose, metabolic, and microenvironmental interactions	Candidate histomorphological feature; direct PCa evidence is currently limited to one retrospective pT3a cohort [12]	No additional stain or molecular assay; spatially explicit assessment of the invasive tumor–adipose interface	Requires adequate sampling of the tumor–adipose boundary; routine biopsy feasibility is unvalidated; no independent PCa validation cohort; reproducibility, incremental prognostic value, clinical utility, and cost-effectiveness remain undetermined [12,24]

## Data Availability

No new data were created or analyzed in this study. Data sharing is not applicable to this article.

## Abbreviations

The following abbreviations are used in this manuscript:

ACAC/ACC	acetyl-CoA carboxylase
ACLY	ATP-citrate lyase
ACOX3	acyl-CoA oxidase 3
ACSM1	acyl-CoA synthetase medium-chain family member 1
ACSM3	acyl-CoA synthetase medium-chain family member 3
ADSC	adipose-derived mesenchymal stem cell
AI	artificial intelligence
AKT	protein kinase B
AMACR	alpha-methylacyl-CoA racemase
AMPK	AMP-activated protein kinase
AR	androgen receptor
AR-V7	androgen receptor splice variant 7
ASCO	American Society of Clinical Oncology
ATGL	adipose triglyceride lipase
BEST	Biomarkers, EndpointS, and other Tools
BMI	body mass index
C/EBPβ	CCAAT/enhancer-binding protein beta
cAMP	cyclic adenosine monophosphate
CCL7	C-C motif chemokine ligand 7
CD3	cluster of differentiation 3
CD8	cluster of differentiation 8
CD9	cluster of differentiation 9
CD36	cluster of differentiation 36 (fatty acid translocase)
CD63	cluster of differentiation 63
CD66b	cluster of differentiation 66b
CD68	cluster of differentiation 68
CD81	cluster of differentiation 81
CHD1	chromodomain helicase DNA-binding protein 1
CI	confidence interval
circRNA	circular RNA
CoA	coenzyme A
CPT1A	carnitine palmitoyltransferase 1A
CRC	colorectal cancer
CRPC	castration-resistant prostate cancer
CT	computed tomography
CTC	circulating tumor cell
CTLA-4	cytotoxic T-lymphocyte-associated protein 4
ctDNA	circulating tumor DNA
DECR1	2,4-dienoyl-CoA reductase 1
DECR2	2,4-dienoyl-CoA reductase 2
DL	deep learning
DNA	deoxyribonucleic acid
EAU	European Association of Urology
EMT	epithelial–mesenchymal transition
ESCRT-I	endosomal sorting complex required for transport I
EV	extracellular vesicle
FABP4	fatty acid-binding protein 4
FABP5	fatty acid-binding protein 5
FASN	fatty acid synthase
FDA	United States Food and Drug Administration
G0S2	G0/G1 switch gene 2
GPX4	glutathione peroxidase 4
H&E	hematoxylin and eosin
HR	hazard ratio
HSL	hormone-sensitive lipase
ICCR	International Collaboration on Cancer Reporting
IGF	insulin-like growth factor
IL-6	interleukin 6
IL-10	interleukin 10
IL-12	interleukin 12
ISUP	International Society of Urological Pathology
lEV	large extracellular vesicle
lncRNA	long non-coding RNA
M1	macrophage with an M1-like phenotype
M2	macrophage with an M2-like phenotype
MAGL	monoacylglycerol lipase
mCRPC	metastatic castration-resistant prostate cancer.
MISEV2023	Minimal Information for Studies of Extracellular Vesicles 2023
miR	microRNA
miRNA	microRNA
mRNA	messenger RNA
MRI	magnetic resonance imaging
mTOR	mechanistic target of rapamycin
NIH	National Institutes of Health
NK	natural killer
PCa	prostate cancer
PD-1	programmed cell death protein 1
PI3K	phosphoinositide 3-kinase
PKA	protein kinase A
PoT	proportion of tumor
PPAR	peroxisome proliferator-activated receptor
PPARγ	peroxisome proliferator-activated receptor gamma
PPAT	periprostatic adipose tissue
PREF-1	preadipocyte factor 1
PSA	prostate-specific antigen
PSMA-PET/CT	prostate-specific membrane antigen positron emission tomography/computed tomography
PTHrP	parathyroid hormone-related protein
pN	pathological regional lymph node category
pT	pathological primary tumor category
*Pten*	phosphatase and tensin homolog
*Rb1*	retinoblastoma 1
REMARK	Reporting Recommendations for Tumor Marker Prognostic Studies
RFS	recurrence-free survival
RNA	ribonucleic acid
RR	relative risk
RSL3	RAS-selective lethal 3
SARIFA	Stroma AReactive Invasion Front Areas
SCD1	stearoyl-CoA desaturase 1
sEV	small extracellular vesicle
SPOP	speckle-type POZ protein
SREBP	sterol regulatory element-binding protein
SREBP1	sterol regulatory element-binding protein 1
SREBP2	sterol regulatory element-binding protein 2
STAT3	signal transducer and activator of transcription 3
TAF	Tumor–Adipose Feature
TCA	tricarboxylic acid cycle
TIGIT	T-cell immunoreceptor with immunoglobulin and ITIM domains
TIM-3	T-cell immunoglobulin and mucin-domain containing protein 3
TNF-α	tumor necrosis factor alpha
TNM	tumor–node–metastasis
*Trp53*	transformation-related protein 53
TSR	tumor–stroma ratio
TWIST1	twist family bHLH transcription factor 1
VEGFA	vascular endothelial growth factor A
